# Supplementary figures and images for: Ovary of Zebrafish during Spawning Season: Ultrastructure and Immunohistochemical Profiles of Sox9 and Myostatin
Source: Animals (Basel). 2023 Oct 29;13(21):3362. doi: 10.3390/ani13213362 (PMC10649070; doi:10.3390/ani13213362)

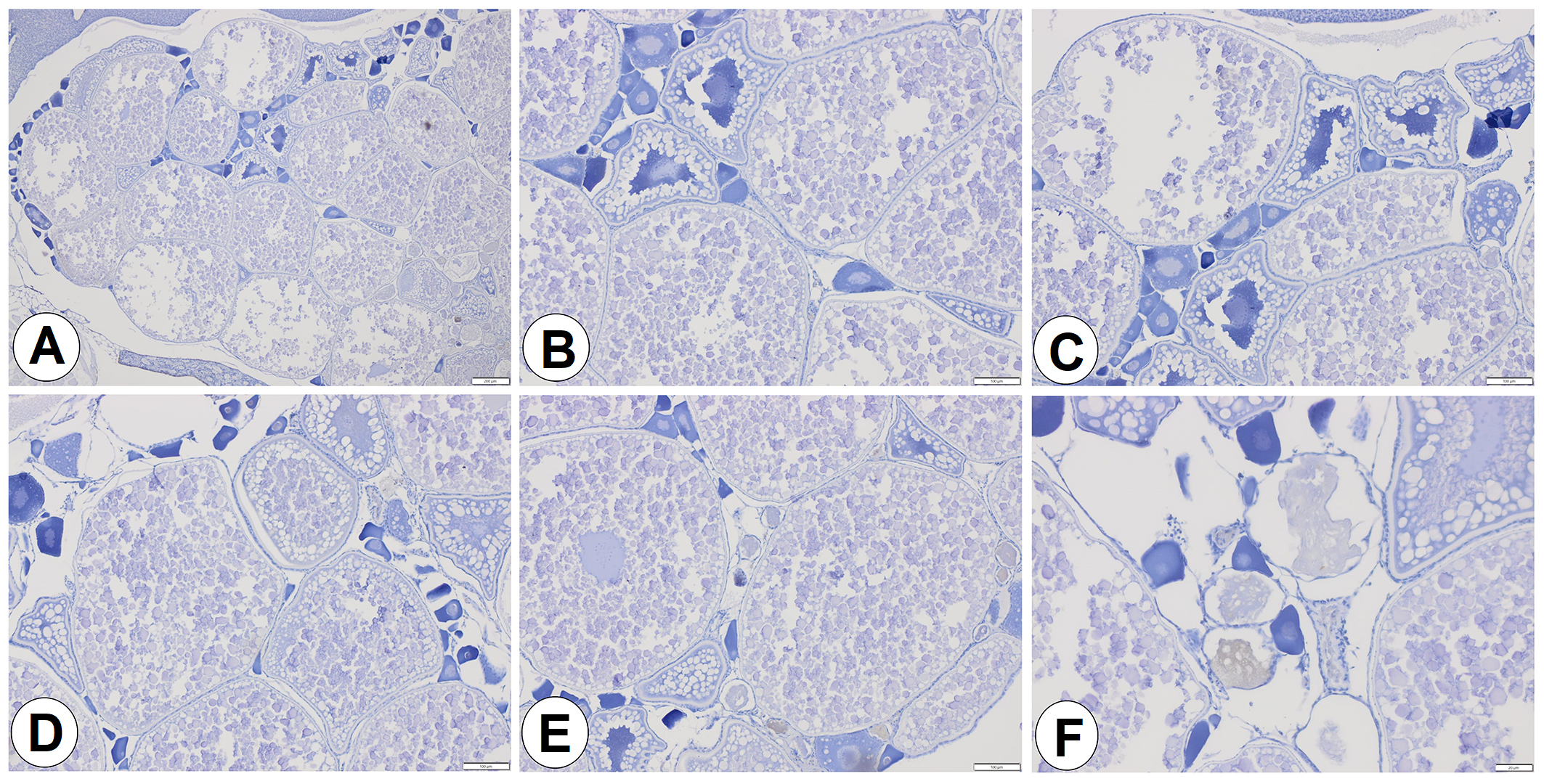

Supplement: Supplementary file 1 [file animals-13-03362-s001.zip › supplementary Figure S1.jpg]
